# Analysis of solid tumor mutation profiles in liquid biopsy

**DOI:** 10.1002/cam4.1791

**Published:** 2018-09-27

**Authors:** Sai A. Balaji, Ashwini Shanmugam, Anuradha Chougule, Srikant Sridharan, Kumar Prabhash, Anuradha Arya, Aditya Chaubey, Arun Hariharan, Pandurang Kolekar, Manimala Sen, Aarthi Ravichandran, Shanmukh Katragadda, Satish Sankaran, Saurabh Bhargava, Prashanth Kulkarni, Suchitra Rao, Chinnababu Sunkavalli, Shripad Banavali, Amit Joshi, Vanita Noronha, Amit Dutt, Urvashi Bahadur, Ramesh Hariharan, Vamsi Veeramachaneni, Vaijayanti Gupta

**Affiliations:** ^1^ Strand Life Sciences Pvt. Ltd. Bangalore Karnataka India; ^2^ Mazumdar Shaw Center for Translational Research, Mazumdar Shaw Medical Foundation Bangalore Karnataka India; ^3^ Tata Memorial Hospital Mumbai Maharashtra India; ^4^ Mazumdar Shaw Medical Center Bangalore Karnataka India; ^5^ Apollo Cancer Specialty Hospitals Hyderabad Telangana India; ^6^ Advanced Centre for Treatment, Research and Education in Cancer Tata Memorial Centre Kharghar, Raigad Maharashtra India

**Keywords:** concordance, ctDNA, monitoring, prognosis, survival outcome

## Abstract

Liquid biopsy is increasingly gaining traction as an alternative to invasive solid tumor biopsies for prognosis, treatment decisions, and disease monitoring. Matched tumor‐plasma samples were collected from 180 patients across different cancers with >90% of the samples below Stage IIIB. Tumors were profiled using next‐generation sequencing (NGS) or quantitative PCR (qPCR), and the mutation status was queried in the matched plasma using digital platforms such as droplet digital PCR (ddCPR) or NGS for concordance. Tumor‐plasma concordance of 82% and 32% was observed in advanced (Stage IIB and above) and early (Stage I to Stage IIA) stage samples, respectively. Interestingly, the overall survival outcomes correlated to presurgical/at‐biopsy ctDNA levels. Baseline ctDNA stratified patients into three categories: (a) high ctDNA correlated with poor survival outcome, (b) undetectable ctDNA with good outcome, and (c) low ctDNA whose outcome was ambiguous. ctDNA could be a powerful tool for therapy decisions and patient management in a large number of cancers across a variety of stages.

## 1 INTRODUCTION

Noninvasive detection and monitoring of disease using circulating tumor DNA (ctDNA) is an active area of research in cancer with considerable implications in clinical management. Liquid biopsies have increasingly become the tool of choice in cases where multiple biopsies are needed for longitudinal monitoring of cancer progression,^1^ detecting emergent drug‐resistance,^2^ evaluating minimal residual disease,^3^ or profiling metastatic tumors.^4^ In addition, liquid biopsy can be an alternative when core and needle biopsies of solid tumors yield DNA of insufficient quantity or poor quality.

To elucidate the clinical utility of ctDNA, it is important to first establish the sensitivity of the technologies being used for its detection through tumor‐plasma concordance studies. Since most concordance studies have heavily relied on advanced and metastatic stages of cancers^2^, ^5^, ^6^ to validate detection technologies, comprehensive data are sporadic in the pre‐metastatic stages across multiple cancer types.^4^, ^7^, ^8^ Given that tumor burden and cell‐free DNA (cfDNA) yields vary depending upon tissue of origin, stage of cancer, ongoing treatment regimen, and other physiological parameters,^4^, ^9^, ^10^ a pan‐cancer study across stages would provide evidence for the widespread applicability of ctDNA as a biomarker.

Research has shown that detection of ctDNA requires digital technologies of high sensitivity. BEAMing PCR, droplet digital PCR (ddPCR), or next‐generation sequencing (NGS) are the platforms of choice to quantify low‐frequency mutant alleles.^4^, ^11^, ^12^ While ddPCR has been shown to reliably identify mutant alleles up to 1 in 1000‐10 000 copies depending upon cfDNA yield, its use is limited to assessing a single or a few mutations simultaneously.^13^, ^14^ For profiling a number of loci across multiple genes, NGS with unique molecular identifiers (UMIs) is preferred. The UMIs distinguish individual DNA copies from PCR duplicates and therefore enhance the limit of detection to 0.5%‐1% allele frequency^15^, ^16^ which is important for ctDNA detection. The best practices with respect to sample handling as well as the standard procedures for each technology are yet to be established.

Studies have correlated cfDNA and ctDNA levels to disease outcomes in patients.^17^, ^18^, ^19^ Some studies have even established the utility of post‐surgery ctDNA levels in predicting disease relapse.^2^, ^20^, ^21^ However, there is no consensus across cancer types and the presurgical levels or levels at biopsy (referred to as baseline levels hereon) of these markers.^7^, ^11^, ^20^

In this study, tumor‐plasma concordance in 180 patients across seven cancer types in the early and the advanced stages was assessed. We saw a concordance of 82% in the advanced stage (Stage IIB and above) cancers and 32% in the early stage (Stage I to Stage IIA) cancers. We observed that ctDNA levels indicated survival outcomes—patients with low (<211 copies/mL plasma) and no detectable baseline ctDNA levels had a survival advantage of 198 and 280 days, respectively. Our data demonstrate that ctDNA can be a reliable marker of the solid tumor in a large number of tissue types in non‐metastatic cancers. Additionally, these presurgical ctDNA can serve a surrogate marker for patient prognosis.

## 2 MATERIALS AND METHODS

### 2.1 Patient recruitment and sample collection

Patients (n = 180) for the study were primarily enrolled from three cancer centers (Table S1) following patient consent and clearance from their respective medical ethics boards. Matched tumor and blood samples were collected from each patient either as fresh tissue or formalin‐fixed, paraffin‐embedded (FFPE) samples at surgery/diagnosis. Ten milliliters of venous blood was collected in Cell‐Free DNA BCT^®^ (Streck, NE, USA).

### 2.2 Tumor DNA extraction and profiling

DNA was isolated from fresh tissue biopsy using QIAamp DNA mini kit (Qiagen, Hilden, Germany) and from FFPE curls using the AllPrep DNA isolation kit (Qiagen). DNA extraction, quantitation, and quality checks were performed as recommended for each panel.^22^, ^23^ Solid tumor DNA was profiled using either the 152‐gene StrandAdvantage (Strand Life Sciences, Bangalore, India) panel (SA152) or the Accel‐Amplicon 56G Oncology Panel v2 (Swift Biosciences, Ann Arbor, MI, USA). Two hundred nanograms was used as input for the SA152 panel while 20‐40 ng was used for the Swift panel. Somatic variants were identified and prioritized using Strand's proprietary tools, Strand NGS (http://www.strand-ngs.com), and StrandOMS (previously StrandOmics), respectively.^22^ Sixty‐four lung cancer samples were tested for *EGFR* mutations on cobas *EGFR* Mutation Test v2 (Roche Molecular Systems, Inc, CA, USA) at source. The maftools package (ver. 1.2.3)^24^ in RStudio (ver. 1.0.136) was used to depict the tumor mutational landscape.

### 2.3 Cell‐free DNA extraction from blood

Following collection of blood in Streck tubes, plasma was isolated within 48‐72 hours of collection. Total cfDNA was extracted from plasma using QIAamp Circulating Nucleic Acid Kit (Qiagen, Cat. No: 55114), as per standard protocol. The genomic DNA contamination in the cfDNA was established using an ALU‐based qPCR assay.^25^

### 2.4 ddPCR

Cell‐free DNA (700‐20,000 genome equivalents) was interrogated for the presence of tumor‐specific mutations using validated ddPCR assays. Droplet generation and PCR were performed using QX200™ Droplet Digital™ PCR System (Bio‐Rad Laboratories, Hercules, CA, USA).^13^ All analyses were performed using the QuantaSoft™ software (v.1.7.4.0917; Bio‐Rad Laboratories) as per the recommendations in the manual.

### 2.5 Liquid biopsy NGS

Cell‐free DNA library preparation was carried out using: (a) Accel‐Amplicon 56G Oncology Panel v2 (without UMIs) with 40 ng of input cfDNA, (b) Human Tumor Actionable Mutations Panel (GeneRead DNAseq Targeted Panels V2; Qiagen; with UMI) with 80 ng of input cfDNA, (c) Rubicon ThruPLEX DNA‐Seq Kit (Rubicon Genomics, Ann Arbor, MI, USA; without UMI), or (d) Rubicon ThruPLEX Tag‐Seq Kit (with UMI) with 20 ng of input cfDNA. Libraries were prepared as per standard instructions from the manufacturers. For the Rubicon kits, the enrichment was performed using the StrandAdvantage 152 gene panel.

For both amplicon panels, the primers were trimmed using cutadapt v1.9.1.^26^ Reads were aligned against the whole genome build hg19 (UCSC). Poor quality reads were filtered as part of QC. For samples prepared on GeneRead, UMI clustering was performed as described by Peng et al^27^ The BAM files were modified with custom scripts to include UMIs in the read IDs to make them compatible with smCounter.^28^ Custom scripts were used to compute average UMI‐depth for each sample and was specified as an input to smCounter to perform variant calling. Since tumor‐specific variants were being queried, we reduced the PI threshold and set a threshold of at least two families of ≥3 reads per family. For the Accel‐Amplicon panel, the Strand^®^ NGS binomial variant caller was used to detect variants in the target regions covered by a minimum of 10 reads, having at least two variant reads and a confidence score of at least 50. Single nucleotide polymorphism (SNP) level quality check (QC) was performed to eliminate false positives. A threshold of 0.3% supporting reads (%SR) was set for SNP detection and 0.2% for insertions and deletions (InDels) spanning multiple bases.

For the Rubicon DNA‐seq, reads that mapped to a particular locus in the genome and had the same alignment start and end positions were assumed to be derived from the same cfDNA molecule and were grouped into single family based on the start and stop positions of each read which served as an UMI. The reads from FASTQ files were aligned against the whole genome as previously described. At known COSMIC loci, reads were grouped into endogenous UMI families with a minimum of 5 reads. A variant was considered bona fide if it was represented by two families of ≥5 reads with >95% of the reads within the family representing it.

In case of the Rubicon Tag‐Seq, FASTQ files were processed according to the manufacturer's instructions using Connor (version 0.5), an open source Bioinformatics tool (https://github.com/umich-brcf-bioinf/Connor). Consensus reads from UMI‐based, positional read families were created using Connor with default parameters. The output BAM file containing the consensus reads was used for SNP detection and downstream analyses using Strand NGS as described above. For tumor‐specific variants, we performed a check for known variants (CKV) and lowered the threshold to 0.4% for SNPs and 0.2% for InDels spanning multiple bases.

### 2.6 Survival analysis

Overall survival (OS) was calculated from the time of enrollment to the clinical endpoint (death) or till the end of the study. The study was carried out for a period of 27 months. Patients reported to be alive were censored at the end of the study or at the date of last follow‐up. The ctDNA level in each sample was represented by the mutation with the highest mutant allele frequency. First, we split the sample set into two categories: where tumor‐specific mutations were detected and where they were not. The sample set where mutations were detected were further divided into ctDNA high and ctDNA low groups based on an optimal cut point of 210.53 copies/mL plasma. The cut point was arrived at using the implementation of the maxstat package within the survminer package in R.

The Kaplan‐Meier estimator^29^ was used to determine the differences in the median survival between the three groups, using a log‐rank test to estimate the statistical significance. The Cox proportional hazard model was used to assess the effect of categorization on overall survival. The survival analysis and the statistical tests performed were implemented using the survminer package (http://www.sthda.com/english/wiki/survminer-r-package-survival-data-analysis-and-visualization) within RStudio (ver. 1.0.136).

## 3 RESULTS

### 3.1 Patient characteristics and study design

**Figure 1:**
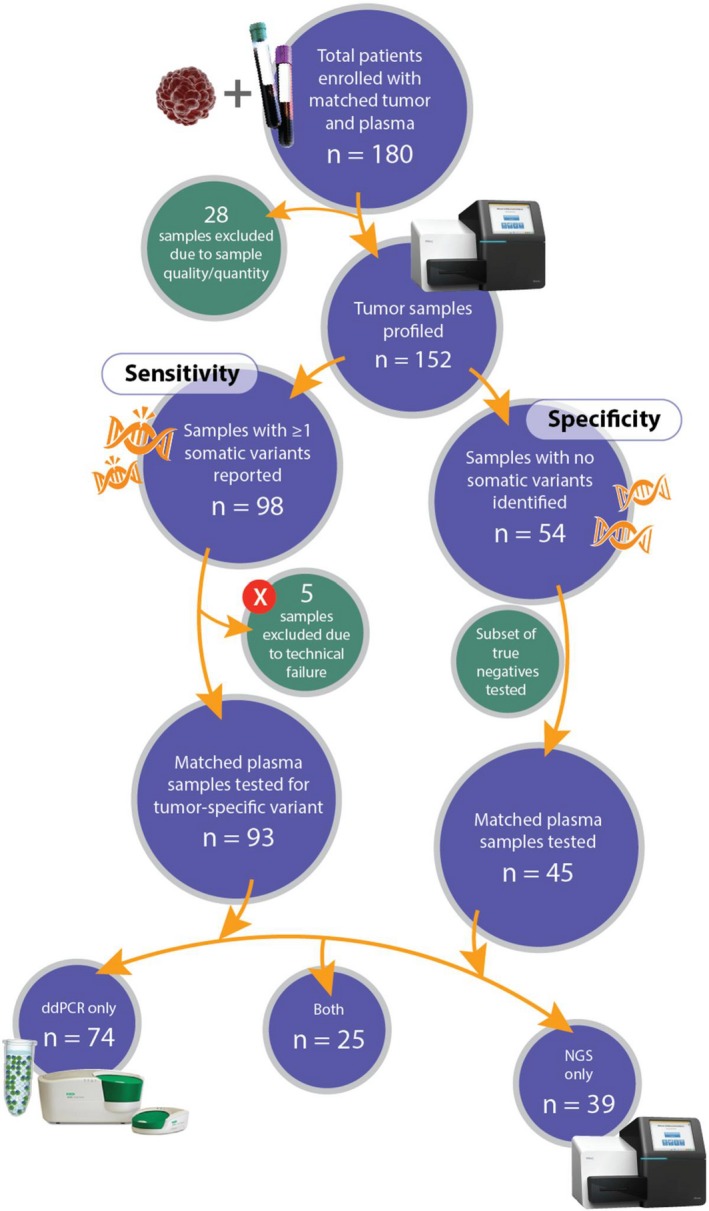
Study design. Matched tumor‐plasma samples were collected from a hundred and eighty cancer patients. Samples were excluded for quality or quantity insufficiency of either tumor or plasma, no reported mutations, or technical failures. The tumor DNA was profiled using targeted NGS sequencing or cobas^®^ *EGFR* Mutation Test. The mutational status of the matched plasma samples were queried by either ddPCR or NGS or both for concordance

One hundred and eighty patients across several different cancer types were enrolled for this study. Lung was the most common site of the primary tumor, followed by breast and colorectal cancers. Other cancers included in the study are bladder, ovarian, esophageal cancers, and sarcoma. The median age at enrollment was 55 years. About 27% of the tumor samples were categorized under Stages I and II by histopathological evaluation as per the American Society of Clinical Oncology (ASCO) staging guidelines (http://www.cancer.net). The stages were classified as follows: (a) Early—upto Stage IIA, (b) Advanced—Stage IIB and above. According to this, 20% of the samples were from early stages, 73.89% were advanced tumors of which 5.67% were metastatic in nature and was undetermined in 6.11%. Patient characteristics are summarized in Table 1. Twenty‐eight samples were excluded from the study either due to poor tissue quality or insufficient sample availability. The schematic of the study design is shown in Figure 1.

**Table 1:** Patient demographics

Patient details	
Total number of patients	180
Age (years)	
Mean (SD)	53.99 (±12.63)
Median (Range)	55 (21‐91)
Unknown (%)	6 (3.33%)
Gender, n (%)	
Female	78 (43.33%)
Male	102 (48.67%)
Tumor type, n (%)	
Bladder	12 (6.67%)
Breast	42 (23.33%)
Colorectal	22 (12.22%)
Esophageal	1 (0.56%)
Lung	93 (51.67%)
Ovarian	9 (5%)
Sarcoma	1 (0.56%)
Clinical stage classification, n (%)	
Early[Link]	36 (20%)
Advanced[Link]	133 (73.89%)
Unknown	11 (6.11%)

Patients with cancer upto Stage IIA are classified as “Early” while patients with cancers which are Stage IIB and above are “Advanced.”

To establish concordance, we tracked the mutational status in the matched plasma in both patients who tested positive and those who negative for tumor type‐specific somatic mutations. We determined the baseline mutational burden in the mutation‐positive patients. We utilized two different types of digital technologies to detect ctDNA in the plasma—ddPCR and NGS. Seventy‐four samples were tested on ddPCR while 39 were run on NGS, and 25 samples were run on both platforms for cross‐platform validation. We tracked survival status in 105 patients over a period of 4‐27 months (Table S2).

### 3.2 Landscape of mutations in the solid tumors

**Figure 2:**
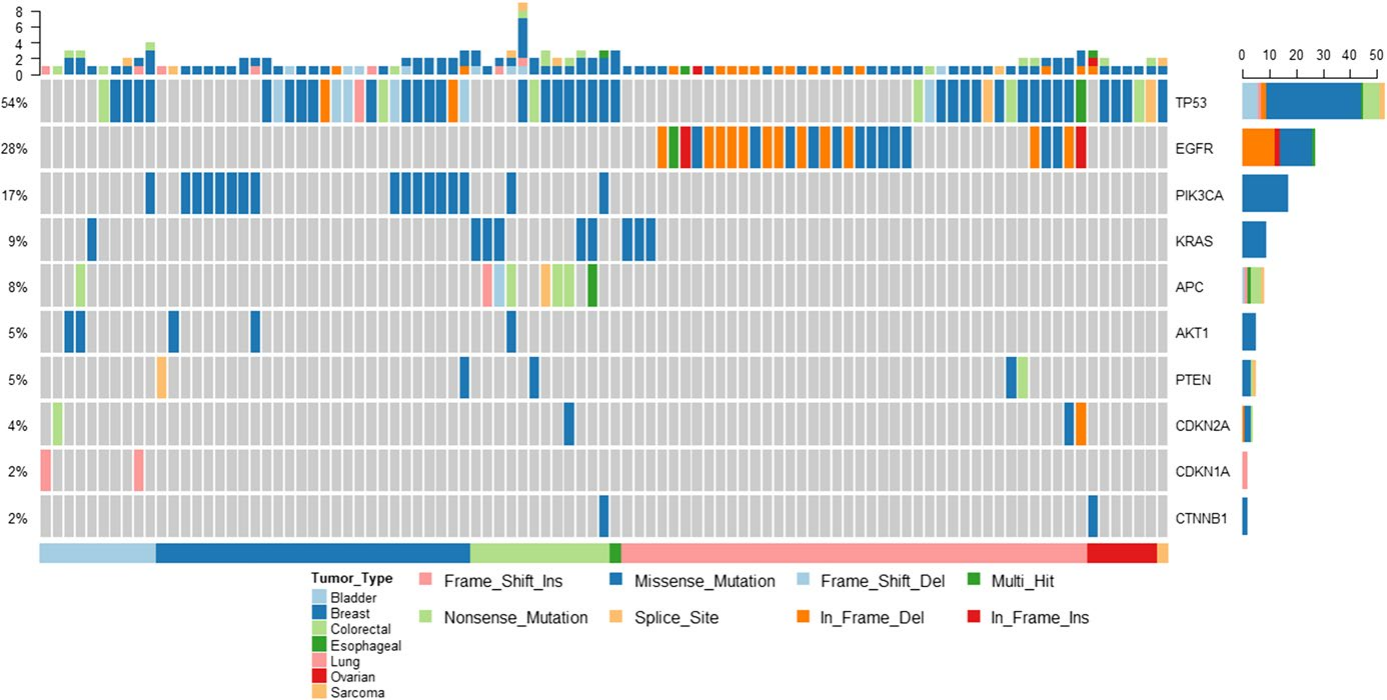
Mutation landscape of tumor samples. A heatmap of mutations across the ten most frequently mutated genes in the tumor samples is shown. Individual samples are depicted along the *X*‐axis while the mutation summary in the gens is indicated along the *Y*‐axis. Samples are colored by tissue types, as indicated by the strip along the *X*‐axis, while the mutations are distinguished by the variant types in the heatmap as indicated in the key. Frequency of mutations per gene is summarized by a histogram along the *Y*‐axis. The samples represented in the heatmap are sorted by their tissue type

At least one tumor type‐specific somatic mutation was identified in 98 tumor samples. Approximately 59.18% samples had only one mutation to follow while around 35.71% reported 2‐3 somatic mutations per sample where a majority was single nucleotide changes (Figure S1A). When the tumor samples were profiled using NGS, *TP53* was the most frequently mutated gene. There were 53 unique mutations identified in the samples, of which only *TP53* p.R175H and *TP53* p.R249S were detected in more than one sample. The tumor mutation landscape is summarized in Figure 2 and Table S3.

To establish concordance between tumor and plasma, samples where somatic mutations had validated TaqMan assays from Bio‐Rad Inc, ddPCR was used to test the matched plasma sample. In 71 samples, at least one somatic mutation detected in the tumor could be followed using a validated ddPCR assay (Table S3). For the rest, NGS technologies, namely SA152, Swift, GeneRead panels, were used depending upon whether the mutation loci were covered.

### 3.3 Performance characteristics of liquid biopsy tests

The yield of cfDNA ranged from 7.13 to 405 ng/mL plasma across all samples. We observed that the yield increased with stage and was significantly higher when compared to that from healthy individuals (Figure S1B). Using a qPCR‐based assay, the mean tumor‐origin DNA fraction in cfDNA samples was estimated to be ~65%. The mean recovery of cfDNA was established at 82%. Both of these parameters were in the expected range.^4^ To establish the precision and recall of ddPCR platform in clinical samples, 68 lung samples with known *EGFR* mutation status for Exon 19 deletions and p.L858R were run. The results matched the expected mutation status for the loci of interest in all except two samples (Table S4), thus showing a precision of 92%. The discordant variant in one sample was confirmed by NGS as a true positive. Recall was established at 100% using the same samples. For precision and accuracy in NGS, we looked at the same two loci, *EGFR* Exon 19 deletions and p.L858R, in all the samples run on the platform (Table S4). There could be a few reasons for the discrepancy between the solid and liquid biopsy in the two discordant samples—(a) the sensitivity of the cobas platform used to determine the EGFR status in solid tumor biopsies is at 1.4%‐5%, or (b) genetic profiling of fine needle aspirate cytology (FNAC) solid tumor biopsies may miss mutations depending upon the site of biopsy due to tumor heterogeneity.

A total of 25 liquid biopsy samples were tested on both ddPCR and NGS, for a cross‐platform comparison. Twenty‐four samples were concordant between the NGS and ddPCR platforms (Table S5). This affirms the thresholds set for detecting known variants. The %SR of the mutations tested ranged from 0.28% to 94.7%. Hence, the platforms show high accuracy, precision, and suitability to test clinical samples.

### 3.4 Evaluation of tumor‐plasma concordance

We report a concordance of 71.2% across all cancer stages and tissue types, irrespective of the technology used (Table 2). For the early stage cancers (≤Stage IIA), the concordance was about 32%, where cfDNA levels are known to be lower^4^ (Figure S1B). For locally advanced and metastatic cancers (≥Stage IIB), tumor‐plasma concordance is 81.8%. We further analyzed our data by each cancer type. Lung cancer samples reported the highest concordance, followed by ovarian and colorectal cancers. The modest concordance observed in bladder and breast cancers could be attributed to the fact that a majority of the bladder samples and at least half of the breast samples were at an early stage. The mutations detected in esophageal cancer and sarcoma were in complete agreement with the solid tumor profile. The concordance data are summarized in Table 2, and the detailed information is provided in Table S6.

**Table 2:** Tumor‐plasma concordance

	Samples	Concordant	Discordant
All samples	138	99 (71.22%)	39 (28.78%)
By stage			
Early (≤Stage IIA)	28	9 (32.14%)	21 (67.86%)
Advanced (≥Stage IIB)	110	90 (81.82%)	20 (18.18%)
By tissue			
Bladder	10	3 (30%)	7 (70%)
Breast	27	10 (37.04%)	17 (62.96%)
Colorectal	11	6 (54.55%)	5 (45.45%)
Lung	81	73 (90.12%)	8 (9.88%)
Ovarian	6	4 (66.67%)	2 (33.33%)
Others[Link]	2	2 (100%)	0 (0%)
By platform			
ddPCR	99	79 (79.8%)	20 (20.20%)
NGS[Link]	64	37 (57.81%)	27 (42.19%)
Cross‐platform Validation			
NGS‐ddPCR	25	24 (96%)	1 (4%)

Includes sarcoma and esophageal.

Results for the three NGS‐based tests, GeneRead, Swift, and the SA152 custom panel.

To call a sample concordant by ddPCR, we set a stringent threshold of 0.03% mutant allele frequency which translates to a minimum of 2 cp/mL plasma given that the median cfDNA yield was ~22 ng/mL plasma. This resulted in an overall concordance of 79.8% for all samples and was as high as 87.05% in the locally advanced and metastatic stages tested on ddPCR. For NGS analysis, we used three different panels. Two commercially available amplicon panels (GeneRead and Swift), and a laboratory‐developed, hybridization‐based enrichment panel, SA152, were used. For the Swift panel, an in‐house noise‐reduction model was utilized to distinguish low‐frequency true variants from false positives. Therefore, irrespective of the presence of UMIs, a minimum of ≥0.3% and ≥0.2% mutant allele frequencies were considered concordant for SNPs and InDels, respectively. Across all NGS panels tested, we report a concordance of 70% for advanced stage tumors and 57.81% overall concordance.

### 3.5 Prognostic value of baseline ctDNA

**Figure 3:**
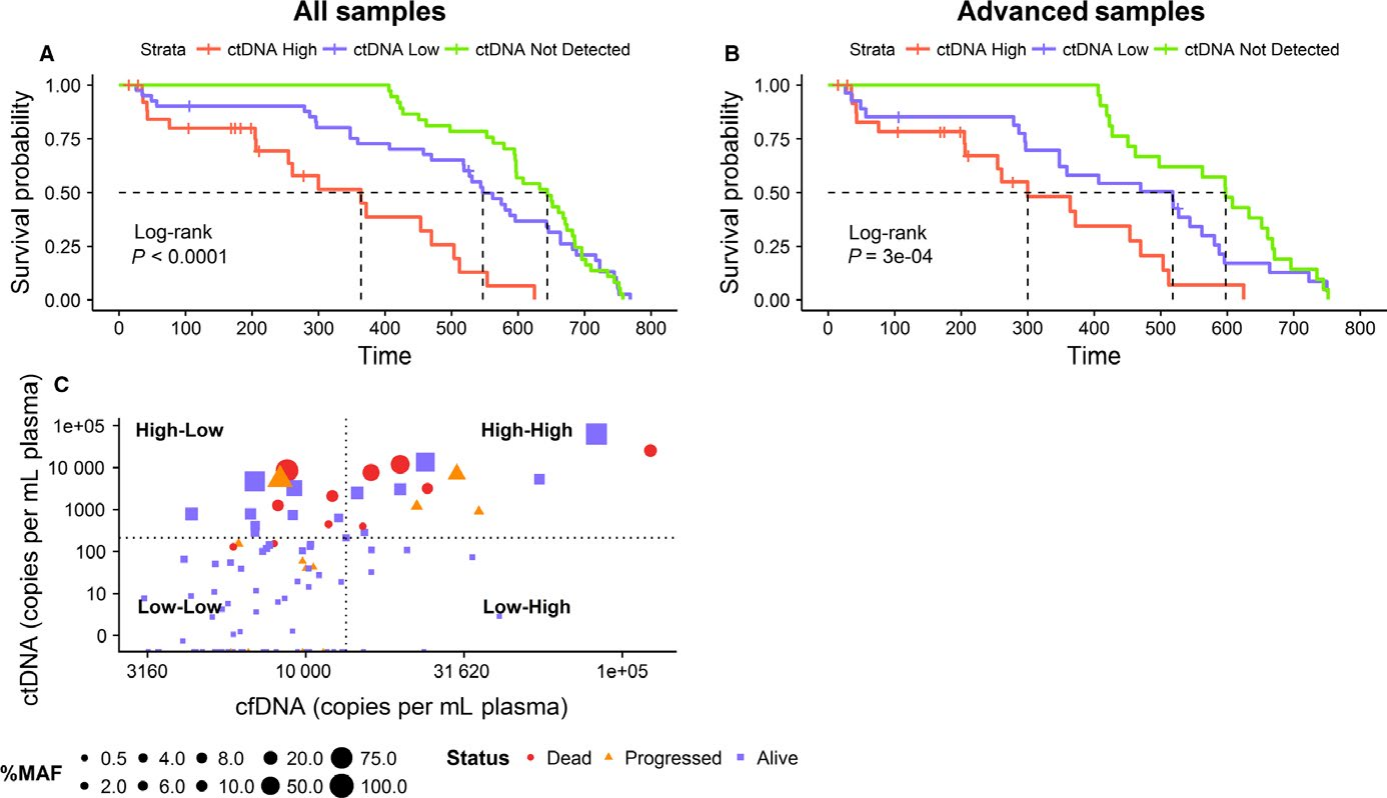
Survival outcomes and baseline ctDNA levels. The Kaplan‐Meier curves indicate the difference in the overall survival of (A) the total cohort and (B) within the advanced stages (Stage IIB and greater). Patients were sorted into three groups: those with high ctDNA (red), low ctDNA (blue), and no detectable ctDNA (green) cp/mL plasma. The median survival of the groups in days is indicated by black dotted lines. The *P*‐values indicated in the graph are estimated using the log‐rank test. (C) Scatter plot indicating ctDNA (*Y*‐axis) versus cfDNA (*X*‐axis) cp/mL plasma. Each point represents one sample. The status of patients is indicated by the color and shape of the point: “Dead” by a red circle, “Progressed” by an orange triangle, and “Alive” by a blue square. The size of each point corresponds to the percent mutant allele frequency (MAF) of the mutation detected in the sample

To understand the clinical significance of the baseline ctDNA levels, we followed 105 patients over a period of about 27 months (Table S2). Our data show that higher baseline ctDNA levels correlate with poor survival irrespective of histopathological stage and tissue type. When partitioned by the number of ctDNA copies/mL plasma, patients with >211 cp/mL plasma (high) show significantly lower survival than those with ≤211 cp/mL plasma (low, *P* = 5.99E‐06) and the undetectable ctDNA (not detected) group (*P*‐value = 2.47E‐07) as shown in the Kaplan‐Meier plot (Figure 3A). Indeed, the undetectable group shows a distinct survival advantage of 280 days compared to the high ctDNA group. The univariate Cox Proportional Hazard Ratio was calculated to be 0.2331 for the low ctDNA group (95% CI: 0.12412‐ 0.4379) and 0.1875 for patients in the undetectable ctDNA group (95% CI: 0.09929‐ 0.3542), therefore indicating a better prognosis (Table S7).

Since most of the patients with poor survival outcomes were from the advanced stages (≥Stage IIB), we repeated the analysis on patients within this group and confirmed the results (*P*‐value = 0.00013 with not detected, *P*‐value = 0.00593 with low, Figure 3B). Interestingly, the same trend holds even in early stage cancers for the ctDNA levels. Of note were two bladder cancer cases, UB002 and UB012, reported as Stage I by histopathological evaluations, where the ctDNA levels were unexpectedly high at 1245.27 and 6947.82 cp/mL plasma compared to the rest of the early stage samples, which reported levels between 0 and 211 copies. Both patients died of disease within the follow‐up period of a year.

To determine whether the absolute levels of baseline cfDNA or percent mutant allele frequency (%MAF) has a bearing on the levels of ctDNA, and therefore survival, we generated a scatter plot to compare the values (Figure 3C). The cutoff value for high and low cfDNA was determined in a fashion similar to identify the ctDNA threshold using the survminer package in R. With respect to %MAF, there is a correlation between the value and ctDNA level as indicated in the graph. The plot shows that patients with >2% MAF did worse than those with lower values. On the other hand, an equal proportion of patients with poor survival and disease progression were categorized under cfDNA high and cfDNA low. The levels of cfDNA could therefore be a less informative marker than either ctDNA or %MAF.

Although there was not a statistically significant difference in the survival outcomes of the low and the undetectable ctDNA groups (*P*‐value = 0.349), there appeared to be a separation of 97 days in the median survival of the two groups. However, there were two patients in the low category who died of disease. In addition, five patients in the ctDNA low group progressed while no patient in the not‐detected category relapsed. Thus, our data suggest that outcomes based on presurgical levels of ctDNA may be definitive for the high and the not‐detected groups (poor and good, respectively). Its predictive value was less clear for the low ctDNA group, where the disease could either progress or report no change. Such patients might benefit from serial monitoring.

## 4 DISCUSSION

Recent studies in liquid biopsy have actively focused on the clinical utility of ctDNA as a surrogate marker in the detection in cancer. A majority of these involved only metastatic cancers,^2^, ^8^, ^16^ while there have been a few that have looked at the early stages.^4^, ^7^ However, most of them have been specific to a cancer type/sub‐type.^21^, ^30^, ^31^, ^32^, ^33^, ^34^ In our cohort, >90% of the patients were non‐metastatic, and spanned across seven different tissue types. Hence, these data provide useful insights into the ctDNA levels across various stages and tissues of origin. The reported concordance is highly variable since it is dependent on the tissue of origin, stage, grade, time of sample collection, and even the platform used for detection.^2^, ^4^, ^6^, ^8^, ^16^, ^19^, ^35^, ^36^ Therefore, our finding of 71.22% overall concordance, 81.82% in stages IIB and above (classified as advanced) and 32% in stages up to IIA (classified as early) is promising. The concordance observed in the early stages gives hope that with some technological advances, early detection, and screening tests may be possible in the near future.

While absolute concordance between tumor and matched plasma has its merit, studies have further explored the clinical utility of ctDNA levels at various time points in the course of the disease and its treatment in patients.^7^, ^11^, ^12^, ^20^ In our cohort, we have observed that the patients’ survival outcomes which strongly correlated to the baseline ctDNA levels. Indeed there appears to be two prognostic groups—those with high ctDNA levels indicative of poor survival, and those with undetected ctDNA who showed good outcome in this period. While the numbers of samples are small, the trends hold even when the data was subset into the early and advanced stages. Though we followed patients for a limited period of 27 months, the trends are stark. Further, the group with detectable ctDNA appears to have a subset with low levels which were less predictive of the survival outcome. These patients might benefit the most from close monitoring to identify relapse before PET‐scans. For those who have undergone surgery, it is possible that post‐surgical MRD levels may be more relevant.^8^, ^11^, ^20^ Equally important, if the patient shows response to therapy, particularly targeted therapy, it will have a higher bearing on the survival compared to any prognostic marker. An example of this is seen in our data, a lung cancer patient with high detected ctDNA (Figure 3C), being treated with Osimertinib upon developing resistance to initial TKI therapy. We found that %MAF and ctDNA levels (in copies/mL plasma) are equivalent prognosticators, with absolute levels of ctDNA stratification showing slightly better statistical significance (Figure S2).

The absolute cutoffs for ctDNA levels may vary across datasets. However, the trend in survival outcomes highlights the clinical relevance of ctDNA levels in prognosis and calls for further research. Studies with larger cohorts may be needed to establish the cutoffs for ctDNA and %MAF in each cancer type for clinical adoption of these biomarkers. In conclusion, our study demonstrates that ctDNA can be used to track tumor‐specific mutations in a large number of cancers reliably and baseline ctDNA levels can be useful markers to stratify patients into prognostic groups which may have a bearing on patient management.

## CONFLICTS OF INTEREST

None declared.
