# Supplementary figures and images for: Analysis of solid tumor mutation profiles in liquid biopsy
Source: Cancer Med. 2018 Sep 27;7(11):5439–47. doi: 10.1002/cam4.1791 (PMC6246960; doi:10.1002/cam4.1791)

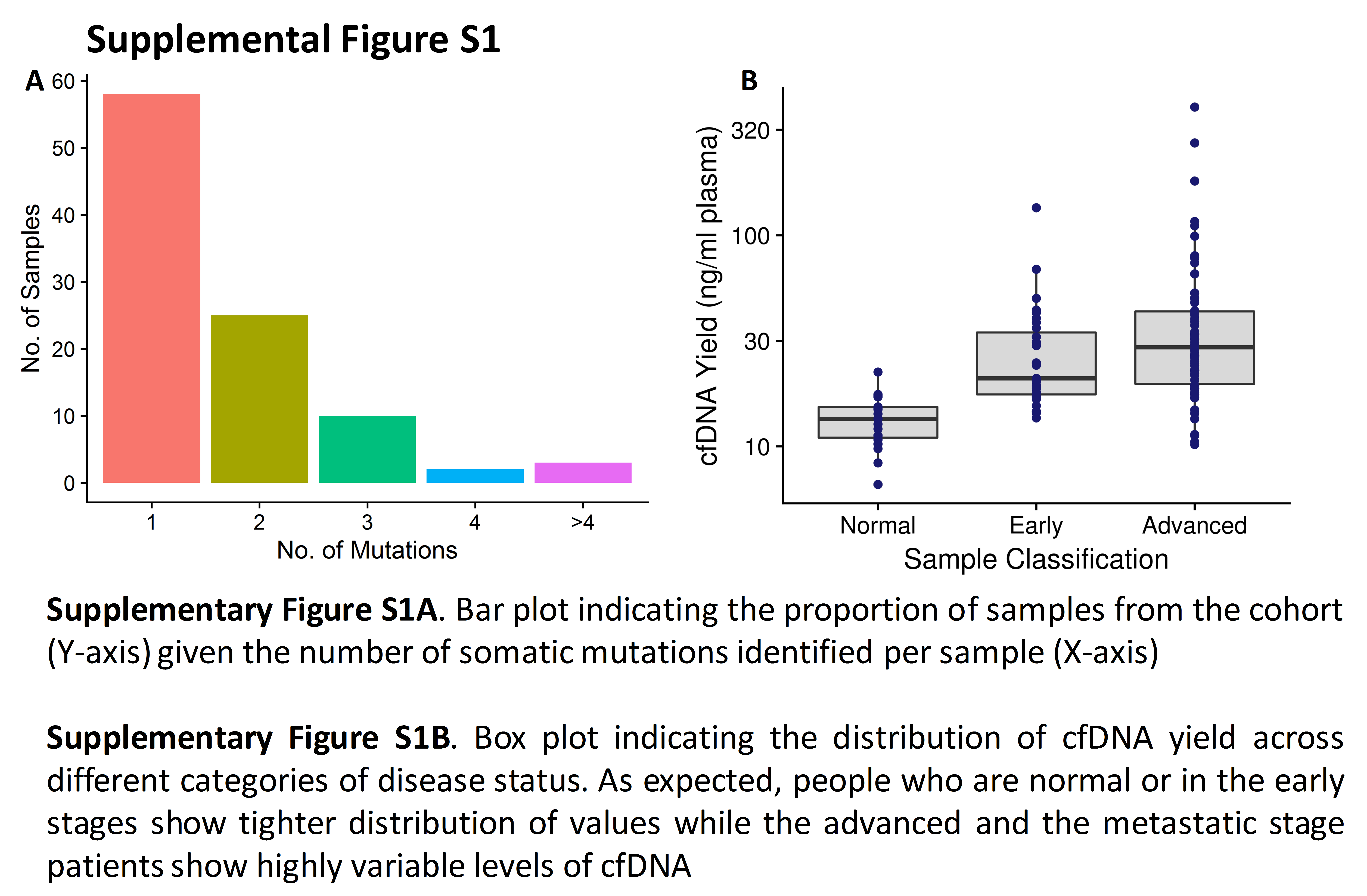

Supplement: Supplementary file 1 [file CAM4-7-5439-s001.png]

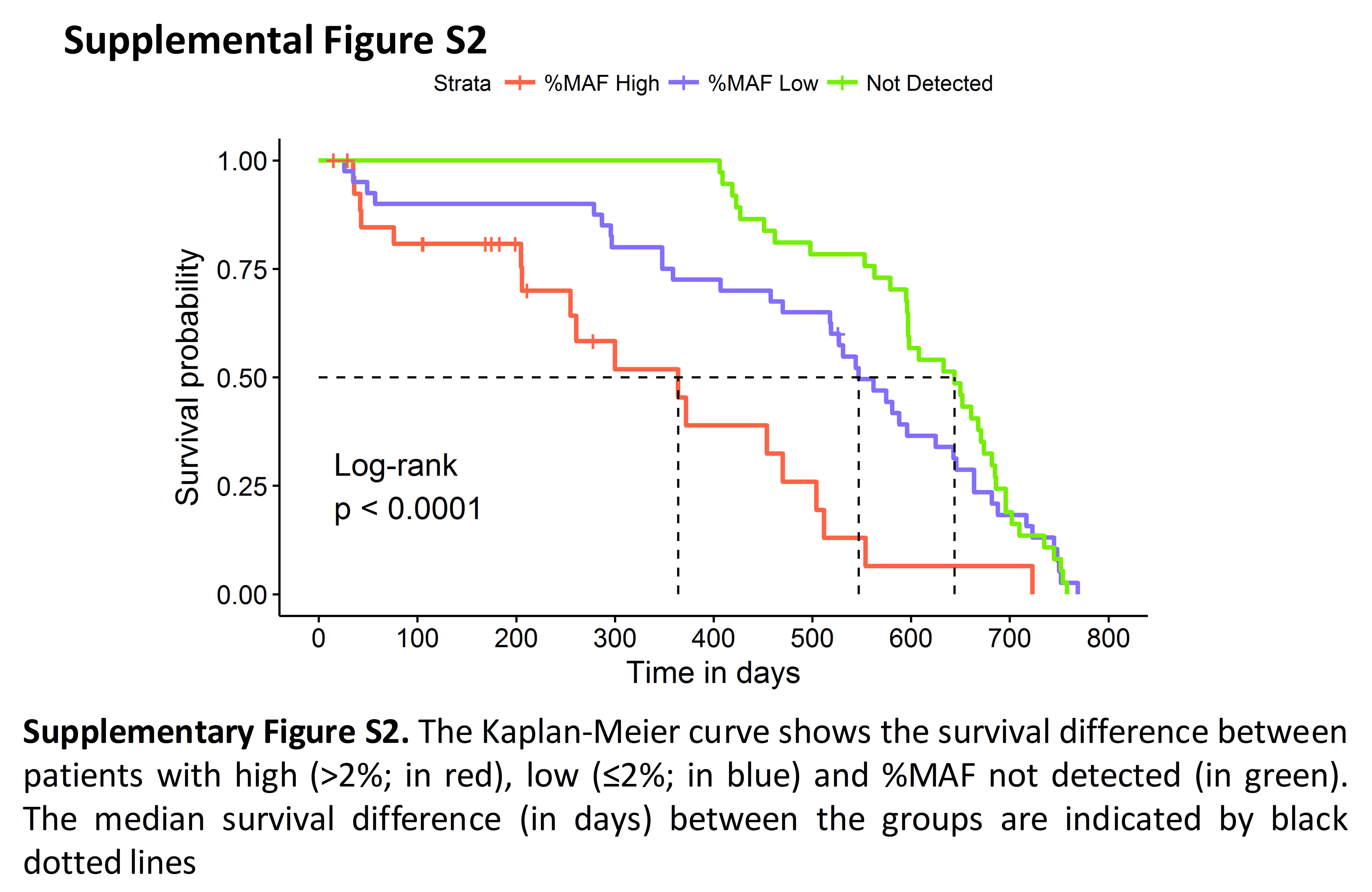

Supplement: Supplementary file 2 [file CAM4-7-5439-s002.png]
